# High Total Hospitalization Cost but Low Cost of Imaging Studies in Recurrent Acute Ischemic Stroke Patients

**DOI:** 10.1371/journal.pone.0101360

**Published:** 2014-07-21

**Authors:** Young Dae Kwon, Sung Sang Yoon, Hyejung Chang

**Affiliations:** 1 Department of Humanities and Social Medicine, College of Medicine and Catholic Institute for Healthcare Management, Catholic University of Korea, Seoul, Korea; 2 Department of Neurology, Kyung Hee University College of Medicine, Seoul, Korea; 3 Department of Health Services Management, Kyung Hee University School of Management, Seoul, Korea; University Of São Paulo, Brazil

## Abstract

**Background:**

Due to the high risk and severity of recurrence after stroke attack, recurrence is a major reason contributing to the disease burden. This study aims to determine whether recurrence is a significant contributor of hospitalization cost in items for ischemic stroke patients.

**Methods:**

This study assessed acute ischemic stroke patients admitted to an academic medical center in 2003 through 2009. The t-test and Chi-square tests were used to compare first-ever and recurrent ischemic stroke groups in terms of total and categorized hospitalization cost, and multiple regression was performed to assess the influence of stroke recurrence.

**Results:**

Recurrent ischemic strokes were associated with higher total cost, but examination cost showed no difference between the two groups. The recurrent stroke group showed higher laboratory but lower imaging cost. Of imaging studies, there was no significant difference in computed tomography scan cost while the first-ever stroke group spent more on magnetic resonance imaging and sonography. Controlling for other influential factors, recurrence was discovered to be a significant factor in lowering examination cost.

**Conclusions:**

The findings of stroke recurrence in lowering examination cost could be explained from two perspectives, different clinical patterns of healthcare utilization and patients' economic status in recurrent stroke.

## Introduction

Stroke recurrence is relatively common. According to the meta-analysis with 13 studies of stroke recurrence, the cumulative risk is 3.1% within the first month, 11.1% within the first year, 26.4% in 5 years, and 39.2% in 10 years [1]. A recent finding from the state-level prospective stroke patient database also showed that about a quarter of all stroke patients suffered a recurrent stroke [2]. Patients with recurrent strokes generally suffer more severe strokes, higher mortality rates, and worsened functional statuses compared to patients with first-ever strokes [3]–[6]. The increasing number of cases of recurrence is the major reason it is becoming the main factor contributing to the disease burden of stroke [7], [8].

Because of the importance of recurrent stroke, there is a considerable amount of research related to recurrence, but the focus has been mostly on the epidemiologic status of recurrence, ways to lower its risk, or ways to prevent it [9]. In practice, clinical practice guidelines for patients with stroke do not refer to special processes of examination and treatment for recurrent patients, except medication changes for patients with antiplatelet history and exclusions of cases of recombinant tissue-type plasminogen activator (rtPA) treatment. The study that compared the costs of first-ever and recurrence treatments concluded that the gap is negligible; making a definite conclusion, however, is difficult due to the age limit of target patients and the limitation of univariate analyses that do not consider other significant influential factors [10], [11].

In order to manage stroke recurrence effectively, it is necessary to understand the clinical differences between first-ever and recurrent patients and provide appropriate treatment and patient care. In this study, the hospital expenses of first-ever stroke and recurrent stroke are compared in total costs and also in categories of resource utilization. The study also includes an analysis to determine whether recurrence has been a significant contributor in itemized hospitalization costs.

## Methods

### Study Design and Subjects

This was a retrospective study that analyzed the hospitalization cost of cerebral infarction patients hospitalized in an academic medical center. The subjects are 1,986 consecutive patients with acute ischemic stroke who were admitted within 7 days of stroke onset to the neurology ward between September 2003 and April 2009. Stroke is defined by the World Health Organization as an event of ‘rapidly developing focal neurological deficits, lasting more than 24 hours or leading to death with no apparent cause other than that of vascular origin' [12]. We defined a recurrent stroke by adding to the above definition the presence of clinical evidence of the sudden onset of a new focal neurological deficit with no apparent cause other than that of vascular origin occurring at any time after the index stroke or the presence of clinical evidence of the sudden onset of an exacerbation of a previous focal neurological deficit with no apparent cause other than that of vascular origin occurring later than 21 days after the index stroke [13]–[15].

The patients were diagnosed based on the results of anamnesis, neurological testing, computed tomography (CT), magnetic resonance imaging (MRI), magnetic resonance angiography, and other neuroradiological findings. The study was approved by the institutional review board of the Kyung Hee University Medical Center, Seoul, Korea. The board permitted a waiver of informed consent because the study involved anonymous data collected for non-research purposes.

### Data and Variables

The data included patients' socio-demographic, hospital access, and clinical characteristics as well as information on hospitalization cost. The data for the first part was collected from a database of the hospital's stroke registry, and the data for the second part was retrieved from the hospital's Patient Management Information. The data for this study will be available upon reasonable request.

The socio-demographic characteristics included age, gender, health insurance type, and risk factors [e.g., prior history of transient ischemic attacks (TIA), smoking, and comorbid conditions such as hypertension, diabetes mellitus, hyperlipidemia, and heart diseases]. The clinical characteristics consisted of referral status, routes of admission and discharge, the severity of each patient's stroke at admission and discharge, the subtype of ischemic stroke, major treatment methods including operation and medication, and length of stay (LOS) at the hospital. These variables were selected based on the fact that, according to previous studies, they directly impact the hospitalization costs of stroke patients.

Each patient's severity, sequelae of previous stroke at admission, and functional outcome at discharge were assessed by certified neurologists. Severity was evaluated by the National Institutes of Health Stroke Scale (NIHSS), and functional outcome was evaluated by the modified Rankin Scale (mRS) difference ( =  admission – discharge). Patients with no records of severity and functional outcome were excluded in the study. With regard to the stroke subtype, each patient was classified according to the Trial of Org 10172 in Acute Stroke Treatment (TOAST) criteria.

With regard to the medical expense data that was collected and analyzed in this study, each patient's hospitalization cost is determined by a fee-for-service schedule based on the type and quantity of services provided. While patients and the National Health Insurance Corporation share the payments for services covered by the National Health Insurance (NHI) or Medical Aid, patients pay in full for the services not covered by these programs. Both insured and uninsured hospitalization costs were included in the analysis.

The various hospitalization costs were categorized into seven items: room/board, laboratory tests, imaging studies, medication, injection, operations and procedures, and others. Six of these seven items were grouped into three broad categories to simplify analyses and facilitate comparisons with previous studies. Those three categories were classified as follows: room and board (as is); laboratory tests and imaging studies (examination); and medication, injection, and operations and procedures (treatment). The “others” category of costs was excluded in the three group classification due to heterogeneous properties of the category.

### Statistical Analysis

Demographic and clinical information was described using frequencies and percentages for categorical variables and means and medians for continuous variables. The proportions of characteristics between first-ever and recurrent stroke groups were compared using Chi-square tests, and the means of total and itemized hospitalization costs between first-ever and recurrent stroke groups were compared using t-tests. Moreover, for each hospitalization cost, multiple regression was developed, which controlled other factors influencing inpatient costs such as the patients' gender, age, treatment methods, LOS, and referral status. The variance inflation factor (VIF) for each predictor for all given cases was computed to detect the existence of multicollinearity. Statistical analyses were performed using SAS 9.1 (SAS Institute, Cary, N.C., USA).

Along with the analysis, inpatient costs were converted to US dollars to enable comparison of the results of this study with those of others, at the exchange rate of US$ 1 = 1,192 Korean won (valid in December 2003). In addition, based on the official medical fee schedules from 2003 to 2009, all monetary values were adjusted to their 2003 equivalents.

## Results

The 1,986 patients with ischemic stroke consisted of 1,595 (80.3%) first-ever stroke patients and 391 (19.7%) patients who had experienced recurrent stroke. The average age of patients with recurrent stroke was significantly higher than that of first-ever stroke patients (64.0 vs. 67.8). Among recurrent stroke patients, markedly high numbers of Medical Aid beneficiaries were noticed. However, there was no significant difference between the two groups in terms of gender. As for risk factors, patients in the recurrent stroke group showed higher rates of hypertension and diabetes but lower rate of tobacco smoking. There was no significant difference between the two groups in terms of TIA, hyperlipidemia, and heart diseases (Table 1).

**Table 1:** Socio-demographic and clinical characteristics of patients.

Variable	Classification	All (n = 1,986)	First-ever (n = 1,595)	Recurrent (n = 391)	Chi-square, *p*-value
		Frequency (%)	Frequency (%)	Frequency (%)	
Gender	Female	785 (39.5)	626 (39.3)	159 (40.7)	0.25, 0.6138
	Male	1,200 (60.5)	968 (60.7)	232 (59.3)	
Health insurance	NHI	1,904 (96.5)	1,541 (97.0)	363 (94.3)	6.57, 0.0103
	Medical Aid	70 (3.5)	48 (3.0)	22 (5.7)	
Risk factors (yes)	TIA	54 (2.7)	41 (2.6)	13(3.3)	0.68, 0.4112
	Hypertension	1,366 (68.8)	1,050 (65.8)	316 (80.8)	32.85, <.0001
	DM	681 (34.3)	507 (31.8)	174 (44.5)	22.53, <.0001
	Hyperlipidemia	677 (34.1)	552 (34.6)	125 (32.0)	0.97, 0.3239
	Smoking	684 (34.4)	584 (36.6)	100 (25.6)	16.95, <.0001
	Heart diseases	375 (18.9)	296 (18.6)	79 (20.2)	0.56, 0.4559
Refer at admission	From other facilities	630 (33.8)	519 (34.8)	111 (29.8)	3.29, 0.0699
Admission route	ER	1,512 (76.3)	1,217 (76.5)	295 (75.6)	0.13, 0.7229
	OPD	469 (23.7)	374 (23.5)	95 (24.4)	
Subtype (TOAST)	LAA	680 (34.2)	537 (33.7)	143 (36.6)	7.00, 0.1358
	SVO	675 (34.0)	557 (34.9)	118 (30.2)	
	CE	185 (9.3)	152 (9.5)	33 (8.4)	
	OD	29 (1.5)	26 (1.6)	3 (0.8)	
	UD	417 (21.0)	323 (20.3)	94 (24.0)	
Treatment (yes)	Operation	43 (2.2)	35 (2.2)	8 (2.0)	0.03, 0.8567
	rtPA	109 (5.5)	92 (5.8)	17 (4.3)	1.22, 0.2692
	Antiplatelet	1,436 (72.3)	1,163 (72.9)	273 (69.8)	1.50, 0.2204
	Heparin	410 (20.6)	316 (19.8)	94 (24.0)	3.43, 0.0641
Refer at discharge	To other facilities	310 (16.6)	228 (14.3)	82 (21.0)	11.10, 0.0009
		Mean (SE)	Mean (SE)	Mean (SE)	t, Pr > |t|
Age		64.79 (0.26)	64.06 (0.30)	67.78 (0.48)	−6.64, <.0001
NIHSS at admission		5.96 (0.13)	5.64 (0.13)	7.28 (0.33)	−4.58, <.0001
mRS difference ( = admission – discharge)		0.37 (0.02)	0.38 (0.02)	0.33 (0.05)	1.08, 0.2815
Length of stay		15.74 (0.41)	14.79 (0.37)	19.62 (1.42)	−3.20, 0.0015

NHI, National Health Insurance; TIA, transient ischemic attack; DM, diabetes mellitus; ER, emergency room; OPD, outpatient department; TOAST, Trial of Org 10172 in Acute Stroke Treatment; LAA, large artery atherosclerosis; SVO, small vessel occlusion; CE, cardioembolism; OD, other determined etiology; UD, undetermined etiology; rtPA, recombinant tissue-type plasminogen activator; NIHSS, National Institute of Health Stroke Scale; mRS, modified Rankin Scale; SE, standard error

Regarding the variables of clinical characteristics, there was no significant difference between the two groups in referral status, admission route, and subtype classification (TOAST). However, our results revealed that patients with recurrent stroke had higher NIHSS and mRS scores, indicating severe neurological impairment and physical disability among recurrent patients. Nevertheless, the difference of mRS scores between admission and discharge was not significant between the two groups. Recurrent stroke patients showed a tendency to have a longer LOS and then transfer to other medical institutions after discharge, but there was no significant difference in the rate of each stroke treatment including surgery, injection, and medication between two groups (Table 1).

The mean total hospitalization cost per patient was $3,752, with daily costs of $289. Among the three broad categories of room and board, examination, and treatment, the average of examination cost ($1,555) was the highest, followed by room and board ($1,177), and then treatment ($906), in descending order. Comparing the two groups of recurrence status, the recurrent stroke group had higher total cost than the first-ever stroke group ($4,423 vs. $3,587), whereas the daily cost was lower in patients with recurrent patients ($270 vs. $294) (Table 2).

**Table 2:** Total, daily, and itemized hospitalization cost of first-ever and recurrent ischemic stroke patients (US dollar).

Variable	All (n = 1,986)	First-ever (n = 1,595)	Recurrent (n = 391)	t-test
	Mean (SE)	Mean (SE)	Mean (SE)	t, Pr > |t|
Total cost	3751.9 (96.4)	3587.4 (78.6)	4422.7 (368.3)	−2.22, 0.0271
Daily cost	289.2 (3.5)	293.8 (4.0)	270.3 (6.7)	3.03, 0.0025
Room and board	1176.7 (40.3)	1100.3 (28.2)	1488.4 (168.7)	−2.27, 0.0237
Examination	1555.2 (20.6)	1552.1 (20.0)	1567.9 (65.3)	−0.23, 0.8181
Laboratory	507.2 (14.9)	475.0 (12.4)	638.8 (55.4)	−2.88, 0.0041
Imaging	1048.0 (10.6)	1077.2 (11.7)	929.1 (23.1)	5.63, <.0001
CT	109.3 (2.8)	109.4 (3.1)	108.7 (6.4)	0.10, 0.9171
MRI	565.7 (6.2)	582.9 ((6.8)	495.4 (14.5)	5.63, <.0001
Sonography	264.0 (3.5)	273.2 (3.8)	226.3 (8.3)	5.35, <.0001
Others	109.0 (5.4)	111.6 (6.3)	98.7 (9.9)	1.10, 0.2714
Treatment	905.5 (42.7)	819.6 (34.7)	1256.0 (163.5)	−2.61, 0.0094
Medication	141.8 (3.7)	133.7 (3.5)	175.0 (12.1)	−3.29, 0.0011
Injection	448.0 (22.6)	411.5 (19.8)	596.7 (81.0)	−2.22, 0.0268
Operation/procedure	315.7 (20.2)	274.4 (16.0)	484.2 (78.4)	−2.62, 0.0091
Others	114.5 (11.4)	115.5 (13.8)	110.5 (13.8)	0.25, 0.8001
Physician fee	38.0 (0.4)	38.1 (0.4)	37.4 (0.9)	0.85, 0.3967
Others	76.5 (11.4)	77.3 (13.8)	73.2 (13.7)	0.21, 0.8312

SE, standard error; CT, computed tomography; MRI, magnetic resonance imaging.

**Figure 1:**
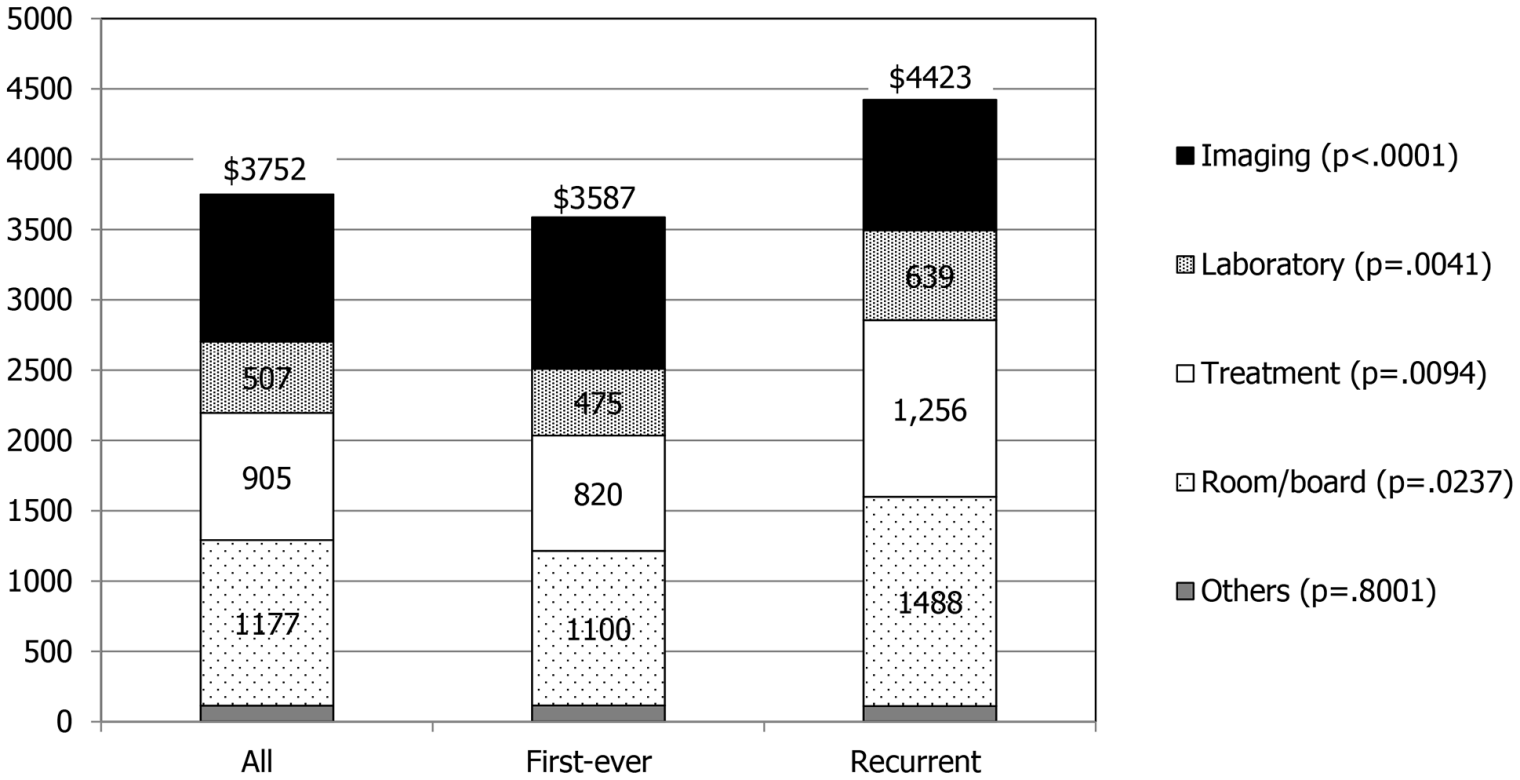
Distribution of hospitalization cost for all, first-ever, and recurrent ischemic stroke patients. The numbers in the bar graph indicate mean hospitalization cost in each category.

**Figure 2:**
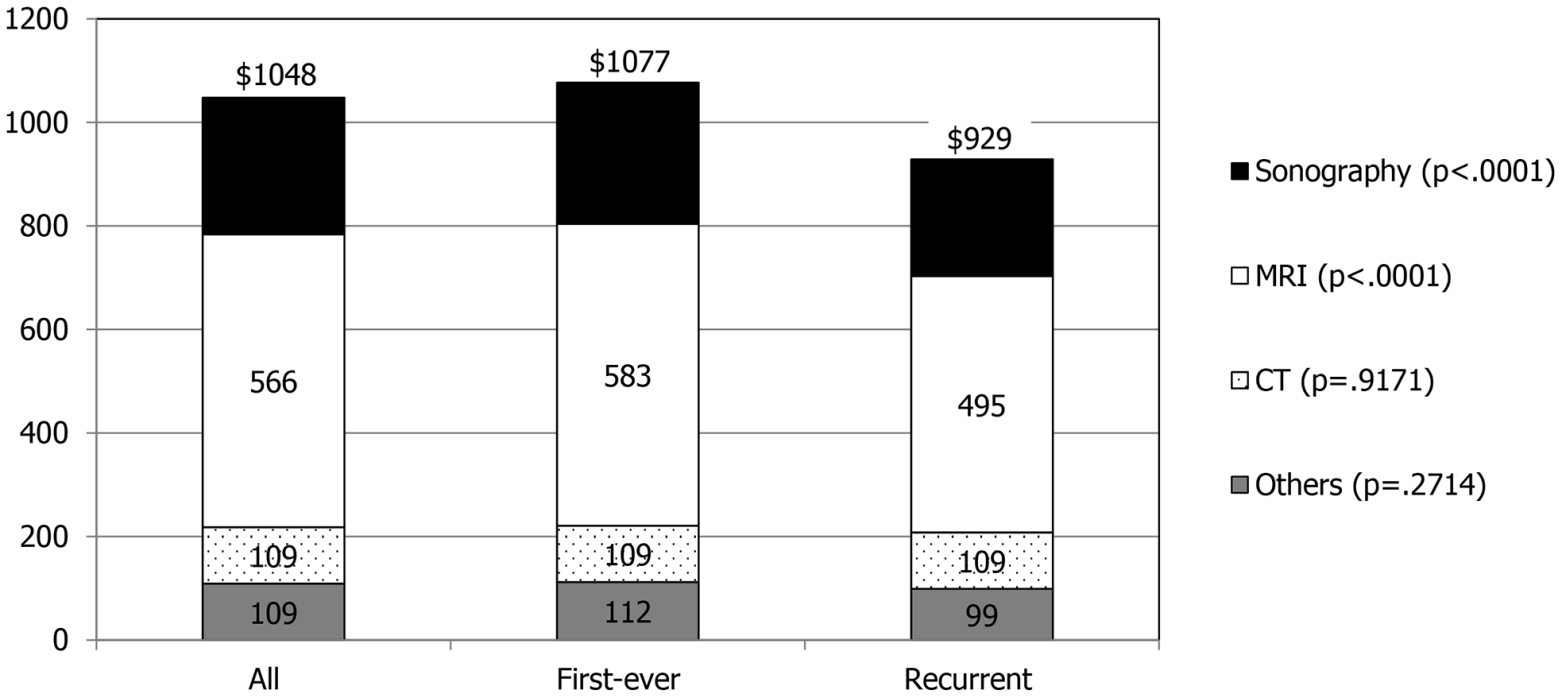
Cost distribution of imaging study for all, first-ever, and recurrent ischemic stroke patients. The numbers in the bar graph indicate mean hospitalization cost in each subcategory.

As for the three categories of costs, the examination cost for stroke showed no difference between the two groups ($1,552 vs. $1,568), while the recurrent group showed higher cost for room and board ($1,488 vs. $1,100) and treatment ($1,256 vs. $820) (Figure 1). However, splitting the examination cost into laboratory and imaging, the recurrent stroke group showed higher laboratory cost ($639 vs. $475) but lower imaging cost ($929 vs. $1,077). Of imaging studies, there was no significant difference in CT scan cost between the two groups while the first-ever stroke group spent more on MRI ($583 vs. $495) and sonography ($273 vs. $226) (Figure 2).

The multiple regression analyses revealed that the significant factors affecting the total cost of ischemic stroke were gender, health insurance type, diabetes, referral status, operation, use of thrombolytics, use of heparin, use of antiplatelet, LOS, transfer to other medical facilities at discharge, and mRS score difference from admission to discharge (Table 3). Recurrence of stroke was not a significant contributor to the total cost of hospital care. However, considering the hospitalization cost by three categories (room and board, examination, treatment cost), the recurrence of stroke significantly influenced examination cost, still not room and board and treatment. An average cost of examination was $90 less in the recurrent ischemic stroke patients (Table 3). Regarding to the VIF for the multiple regression models, there was no indication of the presence of multicollinearity.

**Table 3:** Multiple regression models: impact of recurrence on the total and subcategories of hospitalization cost.

		Total				Room and board				Examination				Treatment			
Variable		Estimate	SE	t value		Estimate	SE	t value		Estimate	SE	t value		Estimate	SE	t value	
Intercept		1236.0	341.0	3.62	^‡^	−335.1	156.1	−2.15	*	1464.3	109.4	13.38	^‡^	123.4	189.4	0.65	
Recurrency	Recurrent	−42.9	126.1	−0.34		30.6	57.7	0.53		−89.8	40.5	−2.22	*	53.9	70.1	0.77	
Gender	Female	−280.7	114.6	−2.45	*	−12.2	52.5	−0.23		−63.8	36.8	−1.73		−150.2	63.7	−2.36	*
Age		−3.0	4.5	−0.67		4.4	2.1	2.13	*	−5.1	1.5	−3.54	^‡^	−2.7	2.5	−1.08	
Health insurance type	Medical Aid	−1015.4	265.4	−3.83	^‡^	−395.0	121.5	−3.25	^†^	−501.4	85.2	−5.89	^‡^	−118.2	147.4	−0.80	
Risk factors	TIA	−2.9	302.4	−0.01		27.2	138.4	0.20		4.8	97.0	0.05		−112.4	168.0	−0.67	
	Hypertension	76.3	110.2	0.69		42.8	50.5	0.85		−0.5	35.4	−0.01		45.6	61.2	0.75	
	DM	219.8	105.1	2.09	*	98.9	48.1	2.06	*	51.3	33.7	1.52		51.9	58.4	0.89	
	Hyperlipidemia	−82.2	104.8	−0.78		3.4	48.0	0.07		−41.5	33.6	−1.23		−26.2	58.2	−0.45	
	Smoking	−41.8	119.5	−0.35		28.5	54.7	0.52		47.7	38.3	1.24		−107.6	66.4	−1.62	
	Heart disease	202.0	179.6	1.12		−34.9	82.2	−0.42		118.3	57.7	2.05	*	−44.5	99.8	−0.45	
NIHSS at admission		10.8	11.1	0.97		−22.0	5.1	−4.31	^‡^	−0.5	3.6	−0.13		32.7	6.2	5.29	^‡^
Subtype (ref: SVO)	LAA	−105.1	122.8	−0.86		−88.5	56.2	−1.58		103.6	39.4	2.63	^†^	−152.3	68.2	−2.23	*
	CE	333.9	247.1	1.35		225.8	113.1	2.00	*	153.1	79.3	1.93		16.6	137.3	0.12	
	OD	−344.5	406.5	−0.85		−77.8	186.1	−0.42		350.4	130.4	2.69	^‡^	−578.0	225.8	−2.56	*
	UD	86.6	146.2	0.59		−74.9	66.9	−1.12		71.4	46.9	1.52		102.4	81.2	1.26	
Admission route	OPD	120.2	117.8	1.02		50.8	53.9	0.94		−9.1	37.8	−0.24		61.9	65.5	0.95	
Referred at admission		−294.5	106.1	−2.77	^†^	19.1	48.6	0.39		−186.0	34.1	−5.46	^‡^	−108.7	59.0	−1.84	
Treatment	Operation	2423.0	338.7	7.15	^‡^	−17.9	155.0	−0.12		738.1	108.7	6.79	^‡^	759.9	188.2	4.04	^‡^
	Thrombolytic	989.6	240.3	4.12	^‡^	505.0	110.0	4.59	^‡^	117.0	77.1	1.52		391.3	133.5	2.93	^†^
	Heparin	−427.2	154.8	−2.76	^†^	−44.1	70.8	−0.62		21.1	49.7	0.43		−411.2	86.0	−4.78	^‡^
	Antiplatelet	−349.8	146.5	−2.39	*	−34.5	67.0	−0.51		−63.5	47.0	−1.35		−298.1	81.4	−3.66	^‡^
Length of stay		203.6	2.9	70.29	^‡^	86.4	1.3	65.13	^‡^	32.3	0.9	34.75	^‡^	79.9	1.6	49.68	^‡^
Referred at discharge		−595.1	148.3	−4.01	^‡^	−89.2	67.9	−1.31		−139.1	47.6	−2.92	^†^	−326.4	82.4	−3.96	^‡^
mRS difference		−343.3	59.4	−5.78	^‡^	−56.6	27.2	−2.08	*	−63.3	19.1	−3.32	^‡^	−176.9	33.0	−5.36	^‡^
Model fit (F-test)		266.84	<.0001			209.92	<.0001			73.9	<.0001			143.19	<.0001		
R-square		0.7821				0.7385				0.4985				0.6583			
Adj. R-square		0.7792				0.7350				0.4918				0.6537			

*P<0.05, ^†^ *P*<0.01, ^‡^ *P*<0.001.

TIA, transient ischemic attack; DM, diabetes mellitus; NIHSS, National Institute of Health Stroke Scale; ref, reference; SVO, small vessel occlusion; LAA, large artery atherosclerosis; CE, cardioembolism; OD, other determined etiology; UD, undetermined etiology; OPD, outpatient department; mRS, modified Rankin Scale.

## Discussion

The purpose of this study was to analyze the differences of hospitalization cost between first-ever and recurrent acute ischemic stroke patients. Previous studies showed that the long-term cost over one year was higher in patients with recurrent stroke than in those who had first-ever stroke but with no difference observed in acute medical cost between the two groups [10], [11]. Our findings revealed that, especially in acute phase, the total cost of hospital care for ischemic stroke was higher in the group of patients with recurrent stroke, compared to those with an initial stroke episode, in univariate comparison. However, the impact of recurrence on total cost was declined in multiple regression analysis after controlling for other factors, because of association of various covariates with one another.

This study showed that the total cost of acute inpatient services was higher in the recurrent stroke group, whereas daily cost was higher in the first-ever stroke group. This finding might have resulted from the fact that patients with recurrent stroke have longer LOS (19.6 vs. 14.8 days). Significantly longer LOS observed in recurrent stroke patients can be interpreted to imply that more serious neurological impairment and physical disability, evidenced by higher scores in NIHSS and mRS, can result in longer LOS. This finding is consistent with the previous finding that people who have experienced recurrence of stroke suffer from high severity of functional status and sequelae following their treatment [6], [10], [16]. It is widely recognized that the level of severity and functional status are significant contributors to increase LOS of stroke patients [17].

Generally, hospitalization cost involves a rapid increase and a sharp decline in the initial phase of hospital admission, followed by a flat period toward the end of hospitalization, resulting in an L-shaped curve [18], [19]. Patients with recurrent stroke tend to spend longer periods in hospitals, resulting in higher total cost. However, the average cost per day decreases due to the lower daily cost toward the end of stay.

We also found that the recurrent stroke group had a higher total cost and higher itemized costs, including room and board as well as treatment; however, interestingly, there was no significant difference in the examination cost between the two groups. The recurrent stroke group had higher cost on laboratory tests but lower cost on imaging studies, resulting in an offset of cost difference, thus causing no difference in the total examination cost between the two groups. However, the difference of examination cost between the two groups became significant in multiple regression analysis, due to other suppressor variables which improve the relationship of recurrence with the examination cost.

In summary, while recurrent patients paid higher cost of room and board, treatment, and laboratory tests directly related to LOS, their cost of imaging studies was lower than those of patients with first-ever stroke. These results can be understood by using two possible hypotheses.

First, from a clinical perspectives, the different pattern of examination or treatment procedures between first-ever and recurrent stroke may cause different estimations of cost. When identifying the underlying diseases or risk factors, the invariable factors may not be tested repeatedly. Moreover, some diagnostic tests and examinations may be skipped for recurrent patients. For example, if there are enough evidences that the subtype of recurrent stroke is identical with that of the initial stroke, the image study may not be necessary to confirm the classification of cerebral infarction. More explicitly, there was no significant difference between the two groups in the cost of brain CT, which is required to distinguish between hemorrhagic and ischemic stroke. However, when the cardioembolic source was confirmed during the first episode of stroke, it was not necessary to repeat the sonography, which consequently decreased the cost of sonography among the patients with recurrent stroke.

Second, from an economic perspective, some tests may be omitted because of the financial difficulty they cause in recurrent patients. Patients who are suffering from substantial economic burdens after the first stroke may refuse more expensive examinations (i.e., imaging studies). This study was not able to measure patient income and purchasing power; thus health insurance type was used to estimate the economic status for the subjects, because economically vulnerable people were designated as Medical Aid beneficiaries. More Medical Aid beneficiaries were found in the recurrent stroke (5.7% vs. 3.0%). Specifically, there was no difference shown between the two groups in the use of CT, the imaging study that was reimbursed by NHI and that required less out-of-pocket payment. However, the patients with recurrent stroke spent less on MRI and sonography studies due to their high out-of-pocket expenses, and these low expenditures support our second hypothesis that patient's socioeconomic status may influence their choice of treatments. In the multiple regression analysis, Medical Aid was a significant factor which affects to decrease both total hospitalization cost and examination cost. Therefore, it is critical to consider patient's economic situation as a possible cause in delaying or precluding appropriate treatment [20], [21]. Further studies need to be conducted in order to analyze the difference between the financial situation of first-ever stroke and recurrent patients and its impact on their treatment and outcome.

A few limitations of this study should be mentioned. This was a single-center study and may not produce results that are generalizable across different patient groups. However, this study did not face the major limiting factors of single-center initiatives, i.e., the difficulty of enrolling a sufficient number or comprehensive group of participants. The study center is a tertiary hospital that plays a major role in the stroke patient community, with its high volume of stroke patients and location in a nationwide catchment area. The research period was relatively long enough to include as many research participants as possible. Although the inflation rate of medical fee was adjusted during the period, other factors which were able to influence on the hospitalization cost were not considered. In addition, the study could not consider the time duration for recurrence, which may throw some additional light on the nature of the costs incurred by the patients, because no information about exact dates of previous strokes was available. The data was collected by the episode of stroke in one hospital, and the only information collected was whether patients had a previous stroke

In spite of these limitations, our study has major implications for clinicians and healthcare policy authorities involved in the redesigning of healthcare payment system. For example, diagnosis-related groups (DRGs) are increasingly being adopted in many countries, even in the care of stroke [22]. The aim of DRG system is to give a concise measure of what hospitals provide to patients and to classify a sufficiently homogenous group of patients. However, prospective payment on the basis of DRGs is likely to limit resources for clinical investigations to ensure fair and appropriate reimbursement through the classification of stroke patients. Quantitative research based on the retrospective cost-based reimbursement such as this study allows researchers to verify whether the most important determinants of cost are considered in patient classification systems. This study suggests that recurrence has the potential to reflect differences in the complexity of treating different groups of patients and to be a factor to classify stroke patients based on healthcare resource consumption.

In summary, our study showed that there were differences in using medical services for the first-ever and recurrent stroke patients during hospitalization. Although the total hospitalization cost in the recurrent stroke group was higher than that in the first-ever stroke group, the examination cost, which included laboratory tests and imaging study, was significantly affected by the recurrence of stroke by lowering the cost. These findings could be explained from two perspectives, different clinical patterns of healthcare utilization and patients' economic situation in recurrent stroke. Further investigations are also suggested to confirm the results of this study in multi-center studies and to analyze the in-depth differences in medical treatments, guidelines, and hospitalization cost between patients with first-ever stroke and patients with recurrent stroke.
